# A highly active nickel electrocatalyst shows excellent selectivity for CO_2_ reduction in acidic media†
†Electronic supplementary information (ESI) available: Including full experimental details, surface coverage measurements and supporting electrochemical measurements. See DOI: 10.1039/c5sc03225c



**DOI:** 10.1039/c5sc03225c

**Published:** 2015-11-24

**Authors:** Gaia Neri, Iain M. Aldous, James J. Walsh, Laurence J. Hardwick, Alexander J. Cowan

**Affiliations:** a Department of Chemistry , Stephenson Institute for Renewable Energy , The University of Liverpool , UK . Email: a.j.cowan@liverpool.ac.uk

## Abstract

The development of selective electrocatalysts for CO_2_ reduction in water offers a sustainable route to carbon based fuels and feedstocks. However, molecular catalysts are typically studied in non-aqueous solvents, in part to avoid competitive H_2_ evolution. [Ni(cyclam)]^2+^ (**1**) is one of the few known electrocatalysts that operate in water and 30 years after its report its activity remains a rarely surpassed benchmark. Here we report that [Ni(cyclam-CO_2_H)]^2+^ (cyclam-CO_2_H = 1,4,8,11-tetraazacyclotetradecane-6-carboxylic acid (**2**)) shows greatly enhanced activity *versus* **1** for CO production. At pHs < p*K*_a_ of the pendant carboxylic acid a large increase in catalytic activity occurs. Remarkably, despite the high proton concentration (pH 2), **2** maintains selectivity for CO_2_ reduction and is believed to be unique in operating selectively in such acidic aqueous solutions.

## Abstract 2

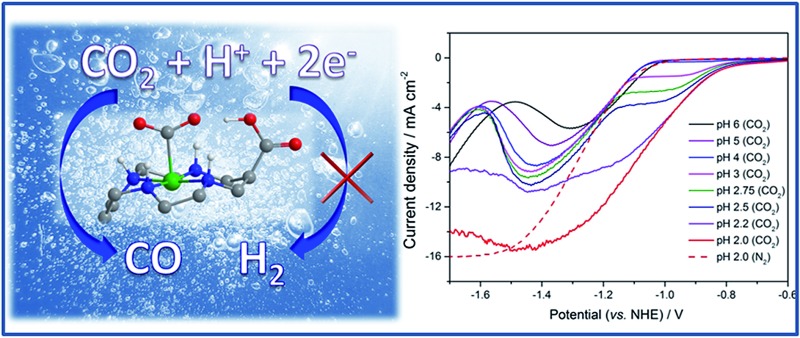
The development of selective electrocatalysts for CO_2_ reduction in water offers a sustainable route to carbon based fuels and feedstocks.

## Introduction

The discovery of catalysts for the conversion of carbon dioxide (CO_2_) into fuels and feedstocks using renewable energy resources such as solar and wind generated electrical is amongst the most significant challenges in chemical research.^1^ Of particular interest is the reduction of CO_2_ to carbon monoxide (CO_2_ + 2e^–^ + 2H^+^ → CO + H_2_O *E*0ap (V_NHE_) = –0.12 – 0.059 pH)^2^ as CO is a key industrial feedstock that can be used to generate a wide range of hydrocarbon products by Fischer–Tropsch chemistry. To enable practical utilisation, CO_2_ reduction electrocatalysts will need to be used in tandem with a sustainable oxidation reaction, such as water splitting (H_2_O → 2e^–^ + 2H^+^ + 1/2O_2_, *E*0ap (V_NHE_) = 1.23–0.059 pH) making the development of low cost, selective CO_2_ reduction catalysts that operate in water at a range of pHs an imperative goal. However the majority of studies to date using molecular catalysts have been carried out in aprotic solvents such as dimethylformamide (DMF) and acetonitrile (CH_3_CN) with Brønsted acids added. Careful control of the acid concentration, coupled to the relatively high solubility of CO_2_ in these solvents minimises competitive H_2_ production (2H^+^ + 2e^–^ → H_2_, *E*0ap (V_NHE_) = 0–0.059 pH). A further complication is that any CO_2_ electrolyser will require the cathode and anode to be separated by a membrane. To date the most effective membranes are proton exchange materials^3^ making the study of CO_2_ reduction in acidic conditions of particular interest. [Ni(cyclam)]^2+^ (**1**) is a low cost, highly selective CO_2_ reduction catalyst producing solely CO in water at pHs 7–4. Since the initial reports over 30 years ago,^4–6^ numerous attempts have been made to develop nickel cyclam catalysts with improved rate constants and onset potentials.^7^ However to the best of our knowledge only two reports observed an increase in the catalyst performance,^8,9^ with functionalisation of both the amines and carbon backbone typically causing losses in selectivity and excessive hydrogen production.

**Fig. 1:**
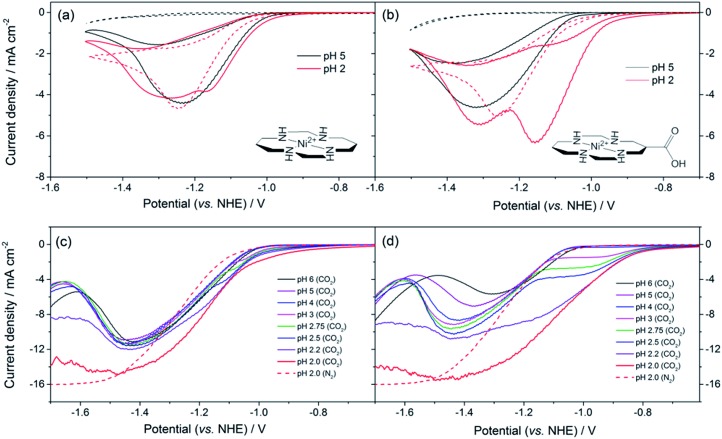
CVs of (a) **1** and (b) **2** (0.1 mM) under CO_2_ (solid lines) and Ar (dashed), at pH 5 (black) and 2 (red). Rotating disk electrode voltammetry of (c) **1** and (d) **2** (0.1 mM) under CO_2_ (solid lines) and Ar (dashed) recorded at 800 rpm at the pH indicated. All experiments are recorded using a Hg–Au amalgam electrode in 0.1 M NaClO_4_.

The mechanism for the reduction of CO_2_ to CO by **1** has been extensively studied,^5,10–13^ and although the exact nature of the active species has yet to be unambiguously identified, several factors have been made clear. Firstly, [Ni(cyclam)]^+^ adsorbs on to some metal electrodes including Sn, Pb,^14^ and Hg,^5^ and adsorption onto the electrode is key for efficient CO_2_ reduction.^11^ **1** has also been shown to act as a homogeneous CO_2_ reduction catalyst when used with a glassy carbon electrode (GCE)^15^ however the level of activity was significantly lower than can be achieved on Hg, which in part may be due to suppression of catalyst degradation pathways on Hg.^16^ Indeed Hg remains a common electrode for fundamental studies such as that presented here. At pH 5 adsorption initiates at potentials positive of the formal Ni^II/I^ couple in solution (–1.3 V_NHE_) and a monolayer is formed at *ca.* –1 V_NHE_. The adsorbed Ni^I^ complex is predicted to bind in an η^1^-C mode to CO_2_, prior to the transfer of a second electron to the catalyst centre.^13^ Computational studies^13,17,18^ indicate that the structure of the adsorbed complex is a *trans*-I conformation,^19^ with the axial amine hydrogens aiding CO_2_ binding. In solution **1** can adopt five different isomeric forms^11^ and for clarity structures of **1** and **2** are drawn in a planar geometry (Fig. 1). Numerous studies have proposed that the decline in activity upon cyclam modification is due to conformational changes limiting the availability of the N–H group suggesting their critical role in catalysis.^5,13,17^ Finally, **1** is most active at pH ∼4–5 indicating that dissolved CO_2_ and not HCO_3_^–^ or CO_3_^2–^ is the preferred substrate. At pH values less than 4H_2_ evolution dominates and CO_2_ selectivity is lost.^9^

Only a limited number of other classes of molecular CO_2_ reduction electrocatalysts for use in water are known.^6,20–25^ Of relevance are recent studies on organic catalysts including mercaptopteridine,^26^ an iridium pincer catalyst^24^ and very recently a water soluble iron porphyrin catalyst, labelled WSCAT,^27^ which preliminary data suggests is an extremely active catalyst at pH 6.7, although at lower pH values only H_2_ was produced. The limited pH range appears to be typical of CO_2_ reduction catalysts with most being studied between pH 6 and 7. In addition to WSCAT, Savéant *et al.* ^28,29^ have also extensively studied other iron porphyrins for use in DMF. In an important breakthrough, a large increase in electrocatalytic activity for CO_2_ reduction to CO by an iron porphyrin modified with phenolic groups in DMF + 2.0 M H_2_O was reported.^22^ The acidic phenol groups on the catalyst framework acted as both a local proton source and to aid CO_2_ binding, greatly accelerating the proton coupled reduction of CO_2_, and similar approaches have now been employed by several groups studying a range of transition metal electrocatalysts for use in non-aqueous solvents.^30–32^ While these studies show that the addition of acidic groups can greatly accelerate the rate of reduction of CO_2_ in DMF, they have not been applied to catalysts that are active in water. Here we demonstrate that the modification of **1** with a carboxylic acid leads to a step change in catalytic activity in water with a five-fold increase in the observed rate constant (*k*_obs_), the turnover frequency per adsorbed catalyst, for **2** compared to **1**, at –0.99 V_NHE_, near the foot of the catalytic wave. We also note an extremely high *k*_obs_ = 3.4 (±1.0) × 10^3^ s^–1^ at –1.25 V_NHE_. Perhaps most remarkable is that catalyst **2** operates in acidic conditions whilst maintaining selectivity towards CO_2_.

## Results

The synthesis of **2**, a derivative of **1** with a carboxylate group on the carbon backbone (Fig. 1) has been reported elsewhere, where we examined the immobilisation of **2** on metal oxide surfaces for the development of a photocatalytic system.^33^ Cyclic voltammograms (CVs) of **1** and **2** on a Hg/Au electrode at pH 5, higher than the p*K*_a_ of the carboxylic acid group of **2** are in line with past reports, Fig. 1a and b. A Ni^II/I^ couple is present under argon at –1.30 V_NHE_ (**1**) and –1.33 V_NHE_ (**2**), (see Fig. S1† for an expansion). Under CO_2_ a large current enhancement, of similar magnitude for both **2** and **1** indicates that catalytic CO_2_ reduction is occuring.^4,33^ At pH 2 under argon the Ni^II/I^ couples of both **1** and **2** are no longer visible by CV, and a catalytic curve due to proton reduction at potentials negative of –1.1 V_NHE_ is observed, Fig. 1a and b. The addition of CO_2_ to **1** at pH 2 leads to only a slight increase in current density, indicating that some CO_2_ reduction may occur at this pH (Fig. 1a), although we show below that H_2_ evolution dominates. In contrast, the current density of **2** under CO_2_ is notably increased and shifted anodic of the current response under argon (Fig. 1b, pH 2) indicating that **2** is an extremely active catalyst for CO_2_ reduction even at very low pHs.

Bulk electrolysis experiments (Fig. S7†) confirm that **2** remains selective towards CO production at pH 2 and that its activity exceeds that of the parent catalyst **1**. 5.2 ± 0.3 C of charge is passed within 1 hour during the electrolysis of an unstirred solution (3 μM) of **2** at –0.99 V_NHE_ with a very good selectivity towards CO production >4 : 1 CO : H_2_ (faradaic efficiency (FE), total = 81%, H_2_ = 15 ± 5%, CO = 66 ± 9%, with errors being the result of 3 experiments). This corresponds to an average bulk turnover number of 591 for CO in 1 hour. In contrast **1** passes only 2.0 ± 0.2C in 1 hour with a lower selectivity 0.2 : 1 CO : H_2_ (FE, total = 86%, H_2_ = 73 ± 16%, CO = 13 ± 10%), and a bulk turnover number of *ca.* 45 for CO production. No liquid phase products were detected by NMR. [Ni(cyclam)]^2+^ and its derivatives are known to form inactive species in the presence of CO,^34^ however activity can be maintained through constant CO_2_ purging and experiments with **2** over a 7.5 hour period show activity being maintained, Fig. S8.†

To understand the factors behind the enhanced activity of **2** at low pH we have examined the electrochemical response of **1** and **2** over a wide pH (6–2) range using rotating disc electrode (RDE) voltammetry, (Fig. 1c and d), differential pulse voltammetry (DPV, Fig. S2 and S3†) and CV measurements (Fig. S4–6†). RDE measurements are employed to study the catalysis under CO_2_ as they minimise the effects of substrate diffusion and product inhibition, simplifying the analysis of the electrochemical response. Between pHs 6 and 4 RDE measurements of **2** under CO_2_ show only a slight increase in plateau current density, Fig. 1d. Between pH 3 and 2 a dramatic change is noted with a new reductive feature (*ca*. –0.95 V) growing in under CO_2_ as the pH decreases, which is shown above to be due to catalytic CO_2_ reduction. This leads to a large decrease in the potential necessary for catalysis between pH 5 and 2 of *ca.* 240 mV *versus* the normal hydrogen electrode. In the RDE measurements we define the potential necessary for catalysis as being when the current density exceeds 2 mA cm^–2^.^35^ In contrast with **1** we only measure a very small shift (*ca.* 50 mV) in the potential necessary for catalysis between pH 6 and 2, which will be at least in part due to the increased level of H_2_ production at low pHs. By pH 2 there is minimal separation of the RDE curves of **1** in the presence and absence of CO_2_, Fig. 1c. This step change in behaviour of **2** but not **1** is indicative of a change in catalytic mechanism for **2** between pH 3 and 2. Furthermore whilst the variation in overpotential for CO_2_ reduction brought about by a change in pH (0.18 V) is equivalent for both catalysts only **2** shows a significant change in potential necessary for catalysis. The lack of a pH dependence for **1** is further explored in the ESI (Fig. S2 and S3†) where we demonstrate that the Ni^II/I^ couple under argon is independent of pH.

**Fig. 2:**
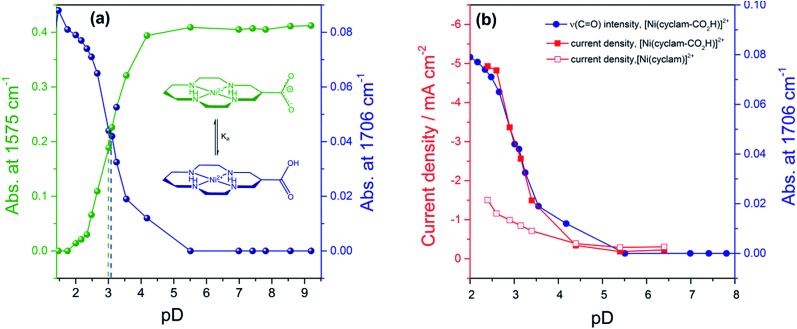
(a) pD titration curves obtained by plotting the IR intensity of the peaks of the carboxylic acid (1706 cm^–1^, blue) and carboxylate (1575 cm^–1^, green). (b) Relationship between CO_2_ reduction current measured at –0.99 V_NHE_ of **2** (filled squares) by rotating disk electrode voltammetry (800 rpm, 100 mV s^–1^), relative concentration of carboxylic acid (given by the *ν*_as_(CO) at 1706 cm^–1^, blue circles) and pD. The current density of **1** under CO_2_ with pH is also shown (open squares). pD = pH + 0.4.

In order to assess if the change in current density under CO_2_ with pH is due to the protonation of the carboxylic acid of **2** we have measured the p*K*_a_ of this group by Fourier-Transform Infrared (FTIR) spectroscopy in solution (Fig. S9†). The spectra were recorded in a 0.1 mm path length CaF_2_ IR cell. The initially synthesised catalyst is prepared in basic conditions and the carboxylate has *ν*_as_(CO_2_^–^) at 1575 cm^–1^ and *ν*_s_(CO_2_^–^) modes at 1375 cm^–1^ in line with literature reports for similar complexes.^36^ Titration of a 0.1 M solution of **2** in D_2_O (initial pD = 9.61) with DCl showed the clear emergence of the carboxylic acid form of **2** with *ν*_as_(CO) at 1706 cm^–1^ in D_2_O, with p*K*_a_ ∼ 2.6. Deuterated solvents are required to avoid the δ(HOH) mode of H_2_O masking the spectral window of interest. There is an excellent correlation between the relative concentration of the protonated carboxylic acid in solution and the current density for **2** measured under CO_2_ using RDE at –0.99 V_NHE_ (Fig. 2b) and –1.1 V_NHE_ (Fig. S10†). This clearly shows that the enhancement in catalytic activity towards CO_2_ reduction is due to the availability of the protonated carboxylic acid group of **2** at low pH values. In contrast a similar pH titration of catalyst **1** shows no clear changes in the spectral and pH region studied.

It has been shown that for **1** the active CO_2_ reduction catalyst is adsorbed onto Hg electrodes.^11^ It is therefore important to ascertain if the active form of **2** is also an adsorbed species. The current density under CO_2_ of **2** on a GCE is found to be significantly lower than that measured on a HMDE (Fig. S11 and S12†) suggesting that the active catalyst is indeed surface adsorbed **2**. The surface concentration of **2** on the HMDE electrode has been measured using double-potential-step chronocoulometry^11^ (Tables S1–S3†) and is found to be 2.0 (±0.2) × 10^–10^ mol cm^–2^ at –0.99 V_NHE_ at pH 5, similar to that previously reported for **1**, 1.6 (±0.2) × 10^–10^ mol cm^–2^.^34^ At pH 2 the surface concentration of both **1** and **2** are changed by a minimal amount (2.2 (±0.2) × 10^–10^ mol cm^–2^ and 1.6 (±0.2) × 10^–10^ mol cm^–2^ respectively at –0.99 V_NHE_), indicating that the large increase in activity of **2** cannot be attributed to a change in the surface coverage of the catalyst with pH.

The kinetic behaviour of **1** and **2** at pH 2 can be obtained from the RDE measurements carried out at different rotation rates (Fig. S15†). We calculate the kinetic activity of the catalyst from voltammetric sweep measurements as it has recently been highlighted that turnover frequencies obtained from long-term bulk electrolysis measurements at high current densities can be complicated by a range of factors including (i) substrate diffusion, (ii) product inhibition and (iii) catalyst deactivation.^37^

Using the limiting current obtained from the intercepts of Koutecký–Levich plots (Fig. S14 and S15†) we can obtain an apparent first order rate constant (*k*_obs_, s^–1^), *i.e.* the turnover frequency (TOF) per adsorbed catalyst using eqn (1).^38^

**Fig. 3:**
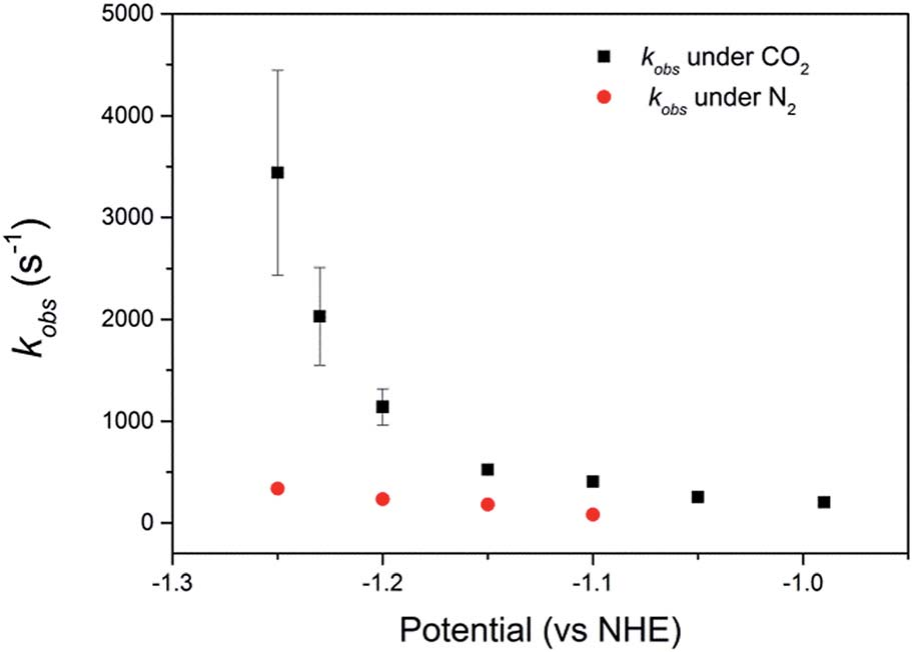
Plot of *k*_obs_ of catalyst **2**, calculated from the intercepts of Koutecký–Levich plots, *vs.* potential at pH 2. Values are obtained averaged from 3 independent measurements with the error bars calculated from the uncertainties in the intercepts of the Koutecký–Levich plots.

1 *i*_cat_ = *nFΓAk*_obs_

where *n* is the number of electrons transferred (2), *Γ* the surface coverage (mol cm^–2^) and *A* the electrode area (cm^2^). We obtain *k*_obs_ values of 3.5 (±1.0) × 10^1^ s^–1^ and 1.9 (±0.2) × 10^2^ s^–1^ for **1** and **2** respectively at pH 2, –0.99 V_NHE_. It is apparent that at pH 2 complex **2** turns over approximately five times faster than **1** at –0.99 V_NHE_ and the activity of **2** exceeds **1** at all potentials examined, Table S4.† It should also be noted that **1** primarily produces H_2_ in bulk electrolysis experiments therefore the measured *k*_obs_ for **1** at pH 2 under CO_2_ is expected to have a significant contribution from proton reduction. In contrast **2** is shown to be selective towards CO_2_ and at potentials positive of –1.3 V_NHE_ there is a large difference in *k*_obs_ obtained in the presence and absence of the substrate (CO_2_), Fig. 3, Table S4.† At potentials corresponding to the plateau current, –1.25 V_NHE_, we calculate a very large rate constant under CO_2_, *k*_obs_ = 3.4 (±1.0) × 10^3^ s^–1^ compared to only *k*_obs_ = 3.3 (±0.4) × 10^2^ s^–1^ under N_2_. This kinetic control between proton and CO_2_ reduction offers a rationalisation of the very high selectivity of **2** even in the presence of a high proton concentration.

## Discussion

Comparison of the catalyst performance with existing benchmarks is ideally carried out by comparison of the overpotential dependence of the catalytic rate constant.^1^ Although these data are becoming increasingly reported for catalysts in aprotic solvents, we are unaware of its availability for the few CO_2_ reduction catalysts that operate in water.^27^ The value of *k*_obs_ = 3.4 (±1.0) × 10^3^ s^–1^ for **2** at pH 2 under CO_2_, measured at a single potential (–1.25 V_NHE_) exceeds the reported TOF of the majority of known water soluble CO_2_ reduction catalysts,^9,24,34^ including **1** (6.3 × 10^1^ s^–1^).^34^ To the best of our knowledge there has only been one reported water soluble catalyst that operates at a greater rate, the recently reported iron porphyrin catalyst WSCAT.^27^ We also note that the measured rate constant for **2** under CO_2_ also exceeds that of many of the most commonly studied CO_2_ reduction catalysts operating in aprotic solvents,^1^ which is perhaps surprising given the significantly lower dissolved CO_2_ concentration in water (0.28 M (CH_3_CN), 34 mM (H_2_O)).

However the most significant feature of **2** is its selectivity towards CO_2_ even under acidic conditions. All previously reported derivatives of **1** have shown predominantly hydrogen production outside of a small pH window^7,11^ and we note that the majority of CO_2_ reduction catalysts are reported at pHs close to neutral (5–7),^24,25,27^ making the ability of **2** to operate at pHs as low as 2 unusual. The correlation between the current density under CO_2_ and the protonation state of the carboxylic acid group of **2** suggests that the protonation state of the catalyst is an important factor in the enhanced TOF, and hence selectivity towards CO_2_ of **2** in acidic solutions (Fig. 2b). It may be envisaged that protonation of the carboxylic acid group leads to **2** being more readily reduced to form the active Ni^I^ catalyst, however DPV studies indicate the Ni^II/I^ couple to be pH independent under argon, Fig. S2 and 3.† Alternatively previous studies have shown that the presence of a local proton source can accelerate CO_2_ reduction and it is viable that the acid group may also aid catalysis here.^28,30–32^ In the homogenous reduction of CO_2_ by **1** in acetonitrile a proton concentration dependent peak in the CV, similar to the feature observed by RDE (Fig. 1d) here at *ca.* –0.95 V_NHE_ was reported.^16^ This peak was assigned to the reduction of a protonated CO_2_ adduct, with this proton dependent electron transfer becoming the rate limiting step in CO_2_ catalysis under certain conditions. It is feasible that the protonated carboxylic acid is acting as local proton source during the reduction of a CO_2_ adduct here. Such an interaction is geometrically feasible. The cobalt analogue of **2** has been reported for use in dye-sensitized solar cells with binding of the –CO_2_H group directly to the metal centre.^36,39^ However we do recognise that the empirical nature of the relationship in Fig. 2b does not provide direct evidence of the functional role of the carboxylic acid. We are currently also unable to discount the role of other potential mechanistic aspects including a possible change in structure of the adsorbed catalyst or change in the catalysts CO_2_ affinity and further mechanistic studies are currently underway.

The enhanced activity of **2** and the ability to maintain selectivity towards CO_2_ across a wide pH range are highly desirable traits. It is likely that robustness towards local pH fluctuations and the ability to be employed in electrolysers using proton exchange membranes will be advantageous for any practically applicable catalyst. However operating at pH 2 does have implications regarding the overpotential for catalysis. The potential necessary for catalysis with a current density of 2 mA cm^–2^ in Fig. 1 is *ca.* –0.9 V_NHE_, corresponding to an overpotential of *ca.* –0.65 V *versus* the apparent equilibrium potential for CO_2_ reduction to CO at pH 2. Whilst not dissimilar to other previous studies in aqueous solutions,^9,27^ it is significantly higher than typically required in solvents such as DMF, CH_3_CN and ionic liquids indicating that further improvements in molecular catalysts for use in aqueous solutions are still required.

## Conclusions

The development of selective and efficient molecular catalysts for electrocatalytic CO_2_ reduction in water is amongst the most challenging goals for the chemistry community. Complex **2** is based on a low cost metal centre and is able to use a pendant acid group to achieve excellent selectivity and activity towards CO_2_ even at the very low pH value of 2. The activity of **2** greatly exceeds the parent complex (**1**) under identical conditions, something that has been rarely achieved in over 30 years of research. **2** is also found to have be amongst the most active aqueous CO_2_ reduction catalysts and we believe that these characteristics make it of great significance to the field of electrocatalytic CO_2_ reduction.
