# Supplementary material for: A highly active nickel electrocatalyst shows excellent selectivity for CO2 reduction in acidic media
Source: Chem Sci. 2015 Nov 24;7(2):1521–6. doi: 10.1039/c5sc03225c (PMC5530941; doi:10.1039/c5sc03225c)
Supplement: Supplementary file 1 [file SC-007-C5SC03225C-s001.pdf]

Electronic Supplementary Material (ESI) for Chemical Science.
This journal is © The Royal Society of Chemistry 2015

A Highly Active Nickel Electrocatalyst shows Excellent Selectivity for CO_2 Reduction in Acidic Media

Gaia Neri, Iain M. Aldous, James J. Walsh, Laurence J. Hardwick and Alexander J. Cowan*

Department of Chemistry, Stephenson Institute for Renewable Energy, The University of Liverpool, UK.

* *a.j.cowan@liverpool.ac.uk*

Electronic supplementary information

Contents

1. Experimental ... 2
   1.1 General ... 2
   1.2 Electrochemistry ... 2
2. Static electrochemistry of 1 and 2 under argon ... 3
   2.1 Cyclic voltammetry ... 3
   2.2 Differential pulse voltammetry ... 3
   2.3 Pourbaix diagrams ... 4
3. pH study of 1 and 2 ... 4
4. Bulk electrolysis ... 6
5. Infrared spectroscopy ... 8
6. Current *vs.* pH plots ... 8
7. CVs of 1 and 2 with HMDE and GCE ... 9
8. Double-potential-step chronocoulometry ... 10
9. Rotating Disk Electrode ... 14
10. References ... 16

1. Experimental

1.1 General

Milli-Q water (18.2 MΩ) was used throughout. $NaClO_4$, NaOH, $HClO_4$, $DClO_4$ and D_2O were used as received (Aldrich). Complexes **1** and **2** were synthesised as previously described.[S1, 2] CO_2, Ar and N_2 were purchased from BOC at CP grade or higher. Gas chromatography was performed using an Agilent 6890N employing N6 helium as the carrier gas (5 ml.min^{-1}). A 5 Å molecular sieve column (ValcoPLOT, 30 m length, 0.53 mm ID) and a pulsed discharge detector (D-3-I-HP, Valco Vici) were employed. CO and H_2 peak areas were quantified with multiple calibrant gas injections and were re-calibrated daily. For FTIR experiments (Bruker Vertex) a CaF_2 IR cell (0.1 mm) was used.

1.2 Electrochemistry

CVs were measured using a Palmsens[3] and a pear-shaped flask with a HMDE (geometric surface area = 0.023 cm^2), Pt coil counter electrode and Ag/AgCl (3 M NaCl) reference electrode (BASi). Experiments were purged with argon, nitrogen or CO_2 for 30 minutes prior to use. The pH was varied by adding $HClO_4$ (1 M) or NaOH (1 M) while keeping the purged solution under a blanket of the relevant gas to ensure a common value between Ar/N_2 and CO_2 experiments. Controlled potential electrolysis (CPE) used a custom mercury pool cell (4.15 cm^2) without stirring or gas bubbling. Prolonged CPE (7.5 hrs) was carried out under a constant CO_2 purge for the first 7 hours, with the faradaic efficiency being determined in the final 30 minutes when the cell is sealed allowing for build-up of products. The counter electrode was separated in a second compartment by a glass frit to minimise re-oxidation of products. Chronocoulometry experiments used a Whistonbrook DF63 potentiostat and Labview software (National Instruments), HMDE working electrode, platinum wire counter and a silver wire quasi-reference electrode. The cell was purged with argon (30 mins) before the experiments and kept under a blanket of argon throughout. A fresh mercury drop was used with every potential step. RDE measurements were performed using a Pine AFMSRCE and Bio-Logic SP-200 potentiostat. The gold electrode (Pine) was dipped in mercury for 1-2 mins, then excess mercury was removed by polishing and left to dry for 2 hours. Amalgam electrodes were found to be stable up to rotation rates of 900 rpm. In the main text we refer to the potential necessary for catalysis, which is defined as the potential when the current density equals 2 mA cm^{-2}. This current density is chosen as it is sufficiently higher than background currents (including currents arising from the initial adsorption of the catalyst).

2. Static electrochemistry of 1 and 2 under argon

2.1 Cyclic voltammetry

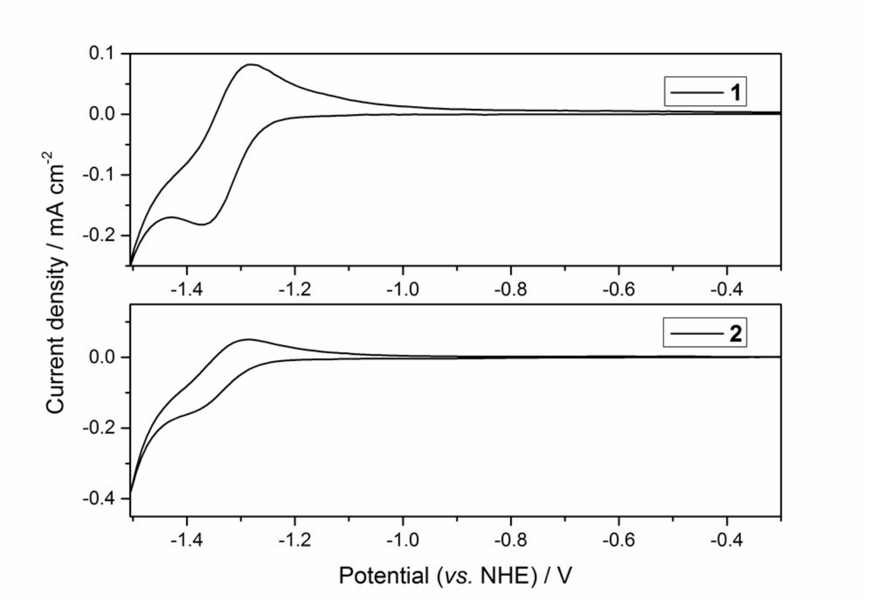

*Fig. S1: CVs of 1 mM **1** and **2** in Ar-purged H_2O containing 0.1 M $NaClO_4$ on a hanging mercury drop electrode (HMDE) working electrode. v = 100 mV s^{-1}.*

2.2 Differential pulse voltammetry

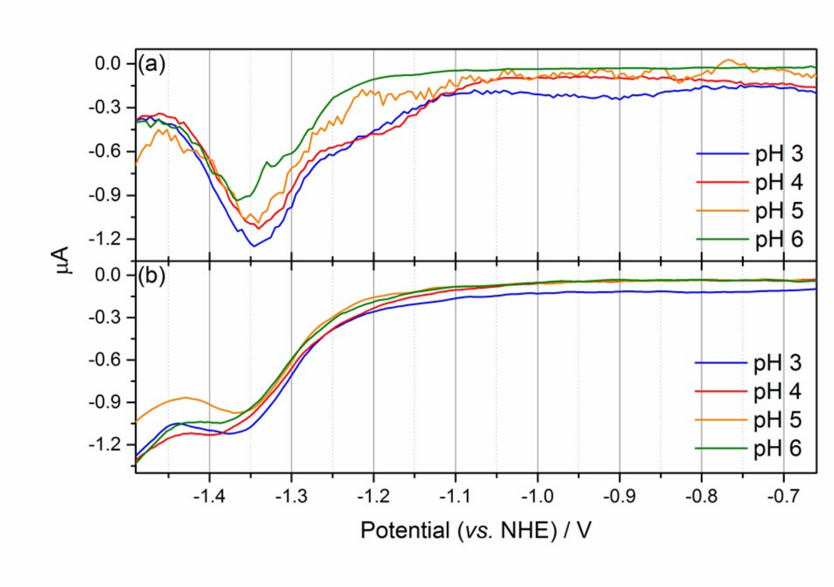

*Fig. S2: Differential pulse voltammetry of 1 mM solutions of **1** (a) and **2** (b) in Ar purged 0.1 M $NaClO_4$ on a gold amalgam electrode at the pHs indicated recorded at 100 mV s^{-1}.*

2.3 Pourbaix diagrams

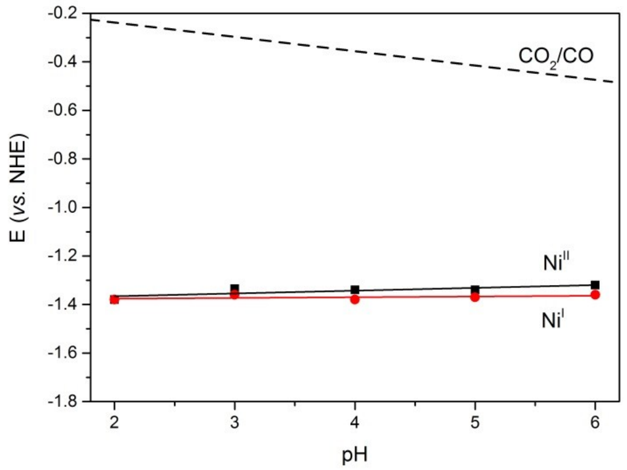

*Fig. S3: Experimental Pourbaix diagram for the $Ni^{II/I}$ couple of **2** (red) and **3** (black)*

The potential of the $Ni^{II/I}$ couple is found to be largely pH independent for both **2** and **1**. In Fig. 2b of the main text we note a correlation between the concentration of the protonated form of **2** and the measured catalytic current density under CO_2. We therefore propose that the change in catalytic activity for **2** with pH is due to the presence of the local acid functionality.

3. pH study of 1 and 2

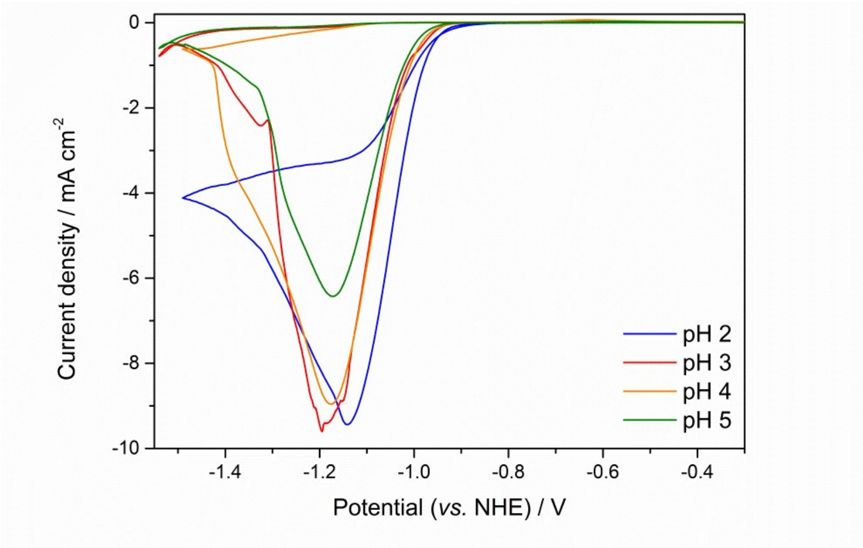

*Fig. S4: CVs of 1 mM solutions of **1** under CO_2 at different pH values, 0.1 M $NaClO_4$, 100 mV s^{-1}, HMDE.*

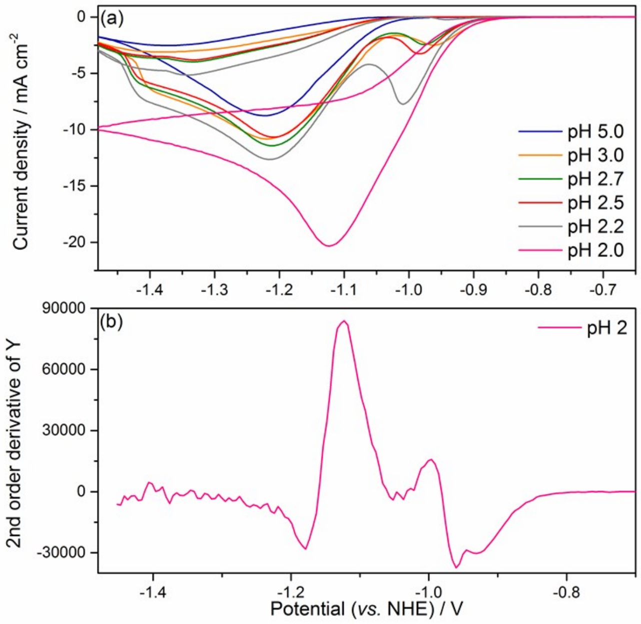

*Fig. S5: (a) CVs of **2** (1 mM) at a range of pH values under CO_2, recorded in 0.1 M $NaClO_4$, scan rate 100 mVs^{-1}, HMDE. (b) Second order derivative of the pH 2 CV under CO_2 for catalyst **2** (forward scan), clearly showing the presence of two reductive peaks.*

The new feature at *ca.* -1 V_{NHE} in Fig S5 is assigned to the presence of the protonated form of the carboxylic acid opening a different mechanism pathway, this peak in the CV corresponds to the feature seen to grow in the RDE in the main text at similar potentials. At pH 2 in the CV the peak at *ca.* -1 V_{NHE} is heavily overlapped with a 2nd reduction at -1.2 V however it can still be identified by taking a second order derivative of the CV (Fig. S5(b)).

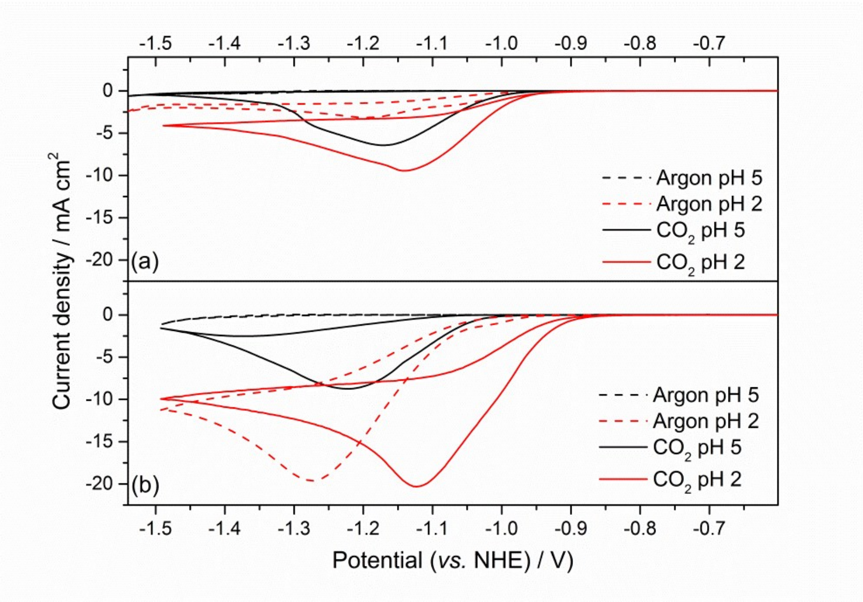

Fig. S6: CVs of (a) ***1*** *and (b)* ***2*** *(1 mM) under* CO_2 *(solid lines) and Ar (dashed), at pH 5 (black) and 2 (red), 0.1 M* $NaClO_4$*, 100 mV* s^{-1} *using a HMDE.*

4. Bulk electrolysis

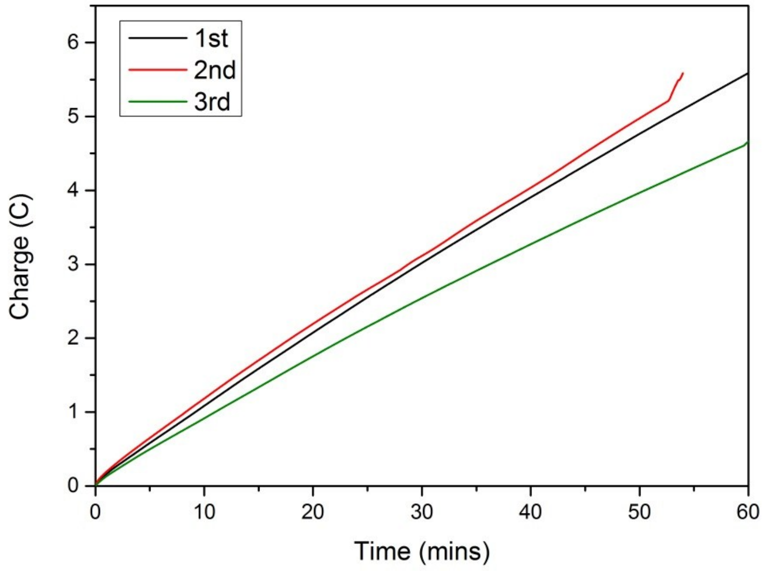

Fig. S7: Consecutive charge vs. time plots for a CO_2*-purged,* $3x10^{-6}$ *M solution of* ***2*** *using a custom made Hg pool electrochemical cell without stirring or gas bubbling (-0.99* V_{NHE}*), 0.1 M* $NaClO_4$*, pH = 2.*

The slight deviation from linearity in the plots is due to CO build up, which is known to deactivate [Ni(cyclam)]$^{2+}$catalysts. Experiments carried out under a constant CO_2 purge at pH 2 using **2** over a 7.5 hour period show reasonable stability with FE for CO = 52 % and H_2 = 31 % being obtained in the final 0.5 of an hour.

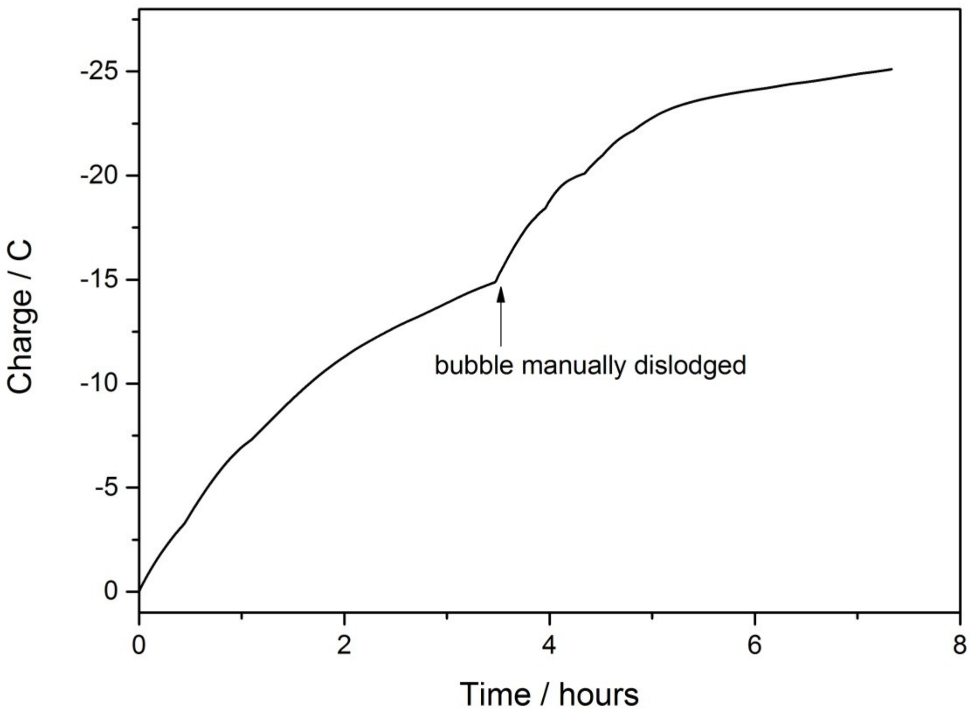

Fig. S8: Prolonged electrolysis of **2** *using a custom made Hg pool electrochemical cell without stirring (-0.99 V_{NHE}), 0.1 M $NaClO_4$, pH = 2. During the final 30 minutes the cell was sealed to allow for the product ratio to be measured. Despite the presence of a constant CO_2 purge large bubbles still formed on the Hg pool electrode during electrolysis.*

5. Infrared spectroscopy

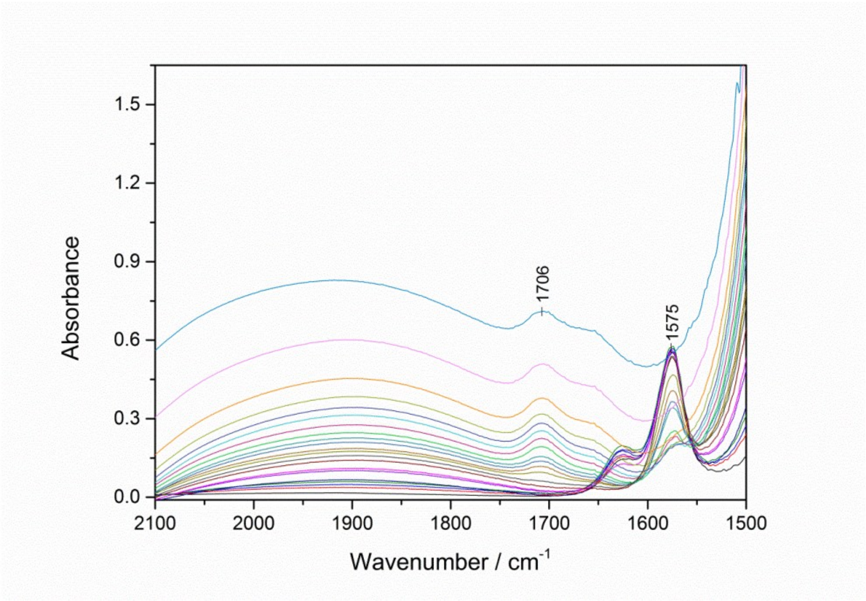

Fig. S9: A series of FTIR spectra for a 0.1 M solution of [Ni(cyclam-CO_2H)]$^{2+}$ in D_2O at decreasing pD values, recorded in a 0.1 mm path length CaF_2 IR cell.

The rising baseline of the FTIR data in Fig. S8 upon addition of D_2O in the region studied (2100-1500 cm^{-1}) is attributed to the presence of HOD, formed due to the presence of a notable concentration of H_2O in the DCl employed.

6. Current *vs.* pH plots

A comparison of the current density recorded at -0.99 V_{NHE} under CO_2 during the RDE measurement at 800 rpm for **1** and **2** with pH is presented in the main text (figure 2b). This potential was chosen as both catalysts are active towards CO_2 and the level of H_2 evolution is also relatively low. A similar trend in current density due to CO_2 reduction with pH is also observed at more negative potentials, Fig S10. Here we have plotted the difference in current density under CO_2 and under N_2 (conditions when only H_2 evolution can occur), to provide an estimate of the current density due to CO_2 reduction only.

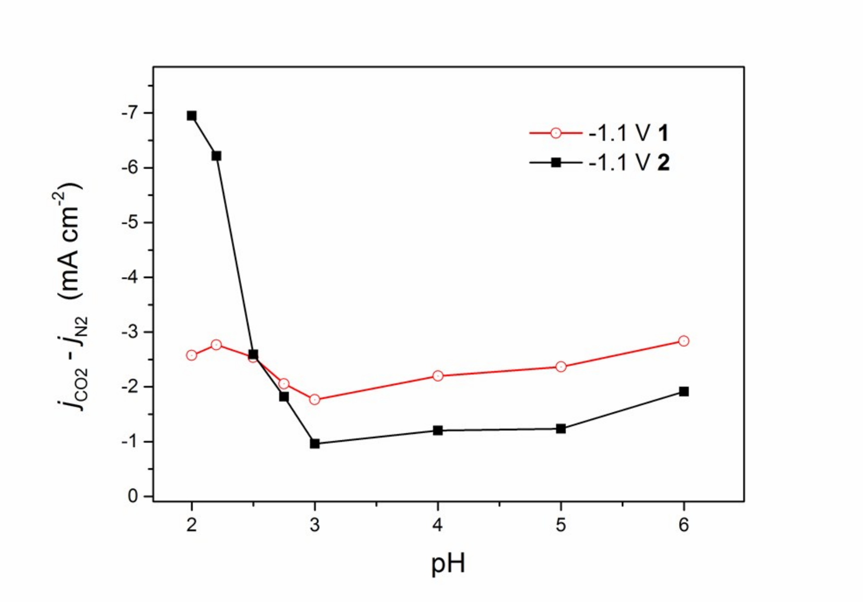

*Fig. S10: Difference between the current density at -1.1 V_{NHE} under N_2 and CO_2 for **1** (red) and **2** (black) plotted versus pH. The current is measured from RDE voltammetry (Au/Hg amalgam working electrode) of 0.1 mM solutions (0.1 M $NaClO_4$) at a rotation of 800 RPM.*

7. CVs of 1 and 2 with HMDE and GCE

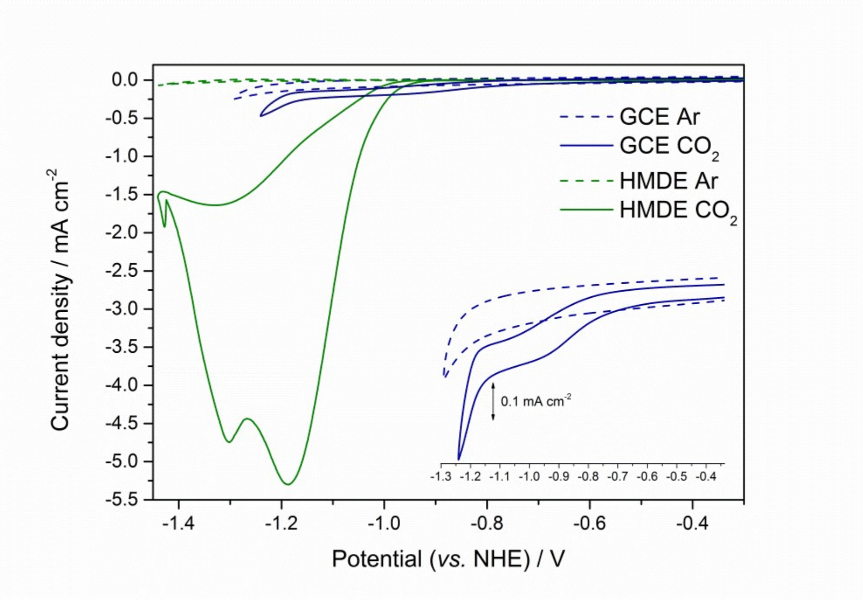

*Fig. S11: CVs of 0.1 mM solutions of **1** recorded with a glassy carbon electrode (GCE) (blue) and a HMDE (green), under Ar (dashed lines) and CO_2 (straight lines), 0.1 M KCl, 100 mV s^{-1}.The inset shows an expansion of the GCE data.*

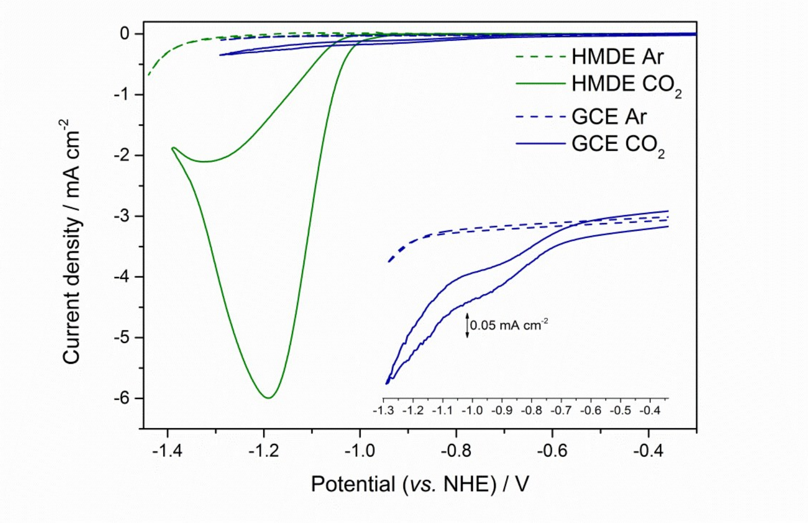

Fig. S12: CVs of 0.1 mM solutions of ***2*** *recorded with a GCE (blue) and a HMDE (green), under Ar (dashed lines) and CO_2 (straight lines), 0.1 M KCl, 100 mV s^{-1}. The inset shows an expansion of the GCE data.*

Experiments at pH 2 were not possible due to the restricted solvent window of the glassy carbon electrode.

8. Double-potential-step chronocoulometry

To evaluate the surface adsorption of **2** to the electrode surface as a function of the potential, double-potential-step chronocoulometry was carried out on 0.1 mM solutions of **2**. The potential was kept at the value of interest for 30 seconds to ensure that the equilibrium quantity of $[Ni^I(cyclam\text{-}CO_2H)]^+$ was adsorbed, then stepped to 0 V for 100 ms and finally stepped back to the initial value. The charge passed during the first 10 ms of the first step was plotted *vs.* $t^{1/2}$, while the charge passed during the first 10 ms of the second step was plotted *vs.* $[\tau^{1/2} + (t- \tau)^{1/2} - t^{1/2}]$, where τ is the duration of the first step, and t is the time. The difference between the forward and reverse intercepts give an estimate of the surface coverage, according to the equation $Q = nFA\Gamma$, where Q is the charge, n the number of electrons, F the Faraday constant, A the area of the electrode and Γ the surface coverage in mol cm^{-2}. However as previously noted by Anson[S3] whilst the reductive desorption is very rapid the time taken for $[Ni(cyclam)]^+$ (and its derivatives) to adsorb is significantly greater than would be expected solely for a diffusion controlled process hence the use of a long

period to achieve equilibrium here (likely required due to a structural isomerisation). This is likely to be a greater cause of error in the application of the measured surface coverages to voltammetric sweep data than the estimated errors obtained from the fit to the intercept of charge *vs.* [$\tau^{1/2} + (t- \tau)^{1/2} - t^{1/2}$] which were typically on the order of 10%.

Table S1: *dependence of the surface coverage of* ***2*** *with the potential for 0.1 mM solutions of catalyst, 0.1 M $NaClO_4$, pH 5. The potential was referenced using the $Ni^{II/I}$ couple.*

**Potential** V_{NHE}	**Surface coverage** mol/cm^2
-1.44	$2.4 \cdot 10^{-10}$
-1.34	$2.6 \cdot 10^{-10}$
-1.24	$2.4 \cdot 10^{-10}$
-1.14	$2.1 \cdot 10^{-10}$
-1.04	$2.3 \cdot 10^{-10}$
-0.94	$2.1 \cdot 10^{-10}$
-0.84	$1.6 \cdot 10^{-10}$
-0.74	$1.6 \cdot 10^{-10}$
-0.64	$1.5 \cdot 10^{-10}$
-0.54	$1.1 \cdot 10^{-10}$
-0.44	$1.1 \cdot 10^{-10}$
-0.34	$0.9 \cdot 10^{-10}$
-0.24	$0.5 \cdot 10^{-10}$
-0.14	$0.3 \cdot 10^{-10}$

Table S2: *dependence of the surface coverage of* ***2*** *with the potential for 0.1 mM solutions of catalyst, 0.1 M $NaClO_4$, pH 2. The potential was referenced using the $Ni^{II/I}$ couple.*

**Potential** $\mathbf{V_{NHE}}$	**Surface coverage** **mol/cm²**
-1.24	$2.2\cdot10^{-10}$
-1.14	$2.1\cdot10^{-10}$
-1.04	$2.1\cdot10^{-10}$
-0.94	$1.6\cdot10^{-10}$
-0.84	$1.5\cdot10^{-10}$
-0.74	$1.5\cdot10^{-10}$
-0.64	$1.2\cdot10^{-10}$
-0.54	$1.3\cdot10^{-10}$
-0.44	$0.8\cdot10^{-10}$
-0.34	$0.4\cdot10^{-10}$
-0.24	$0.4\cdot10^{-10}$
-0.14	$0.2\cdot10^{-10}$

Table S3 *dependence of the surface coverage of* ***1*** *with the potential for 0.1 mM solutions of catalyst, 0.1 M $NaClO_4$, pH 2. The potential was referenced using the $Ni^{II/I}$ couple.*

**Potential** $\mathbf{V_{NHE}}$	**Surface coverage** **mol/cm²**
-1.14	$2.2\cdot10^{-10}$
-1.04	$2.3\cdot10^{-10}$
-0.94	$2.2\cdot10^{-10}$
-0.84	$1.9\cdot10^{-10}$
-0.74	$1.2\cdot10^{-10}$
-0.64	$1.5\cdot10^{-10}$
-0.54	$1.3\ 10^{-10}$
-0.34	$0.7\cdot10^{-10}$
-0.24	$0.3\cdot10^{-10}$
-0.14	$0.1\cdot10^{-10}$

9. Rotating Disk Electrode

Analysis of RDE data allows for calculated k_{obs} in the absence of diffusion control. Rotation rates of up to 900 rpm were achieved with no noticeable disruption of the amalgam coating. The limiting currents are calculated from the intercept of the Koutecký-Levich plots, constructed using three independent measurements, a representative example of which is provided in Fig. S15. From these currents we calculate k_{obs} using Eq 1. in the main text in the manner recently described elsewhere.[S7,8] Here k_{obs} describes the overall rate law for the system as the complete mechanism remains unproven. We note that the mechanism of CO_2 reduction by **1** and its derivatives has been proposed to involve initial reduction of Ni^{II} followed by reaction with CO_2, with a 2nd electron transfer and chemical step following.[S4-6] We also propose that with **2** reduction to Ni^{I} occurs prior to CO_2 binding as UV/Vis studies show no interaction between the Ni^{II} and CO_2, such a process would lead to change of coordination environment from square planar for **2**, which would lead to a clear change in the UV/Vis spectrum[S2] (Fig. S13)

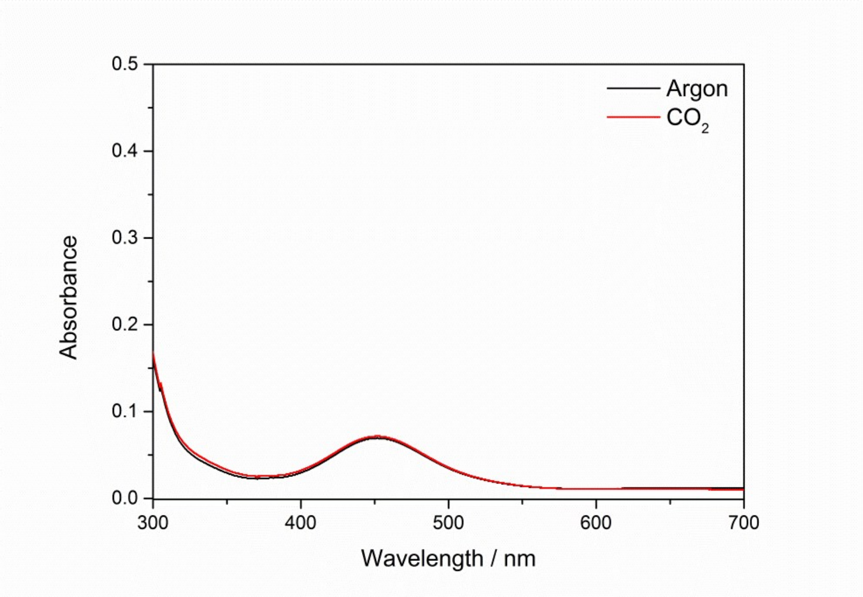

*Fig. S13: UV-vis spectra of **2** (3 mM) at pH 2 under argon (black) and CO_2 (red), 0.1 M $NaClO_4$, showing no change in the Ni^{II} coordination geometry.*

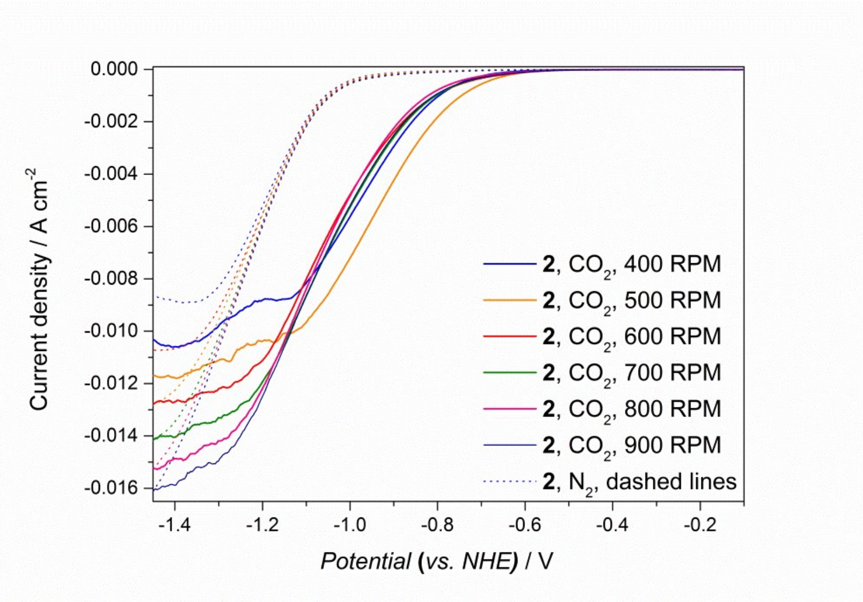

Fig. S14: Rotating disk electrode voltammetry of **2** *(0.1 mM) under nitrogen (dashed) and CO-${}_2$ (solid lines), 100 mV s^{-1}, 0.1 M NaClO$_4$, pH 2, recorded using a Hg-Au amalgam electrode.*

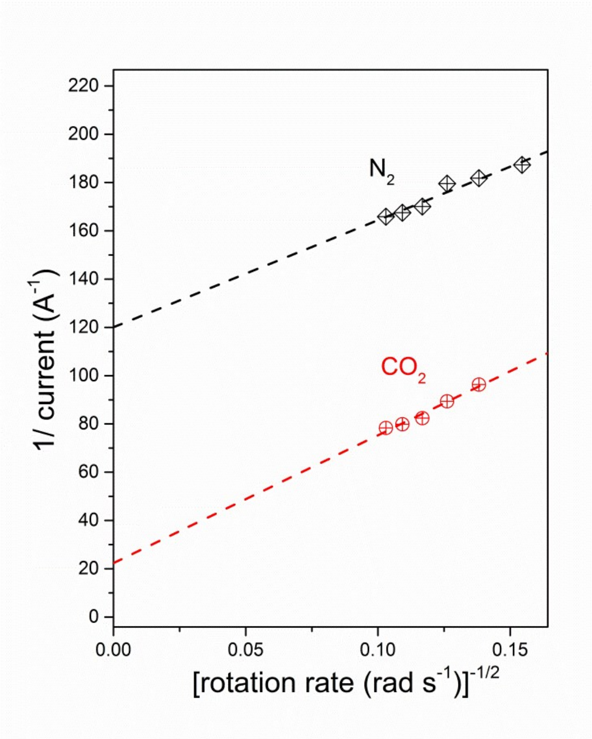

Fig. S15: Example of a Koutecký-Levich plot for **2** *(0.1 mM, 0.1M NaClO$_4$), under argon and CO$_2$, calculated at -1.25 V$_{NHE}$.*

V_{NHE}	k_{obs} CO_2 (s^{-1}) x10^2	Error ± (s^{-1}) x10^2	k_{obs} N_2 (s^{-1}) x10^2	Error ± (s^{-1}) x10^2

**Catalyst 2**				
-0.99	2.02	0.28	NA	
-1.05	2.54	0.27	NA	
-1.10	4.05	0.25	0.81	0.16
-1.15	5.23	0.45	1.81	0.14
-1.20	11.39	1.75	2.34	0.16
-1.23	20.30	4.80		
-1.25	34.39	10.02	3.38	0.38
**Catalyst 1**				
-0.99	0.35	0.10	0.04	0.01
-1.10	1.39	0.45	0.53	0.05

*Table S4: k_{obs} for catalysts **1** and **2** at different potentials, calculated from Koutecký-Levich plots, and relative errors. At potential below -1.1 V_{NHE} using catalyst **2** the value of k_{obs} measured under N_2 was found to be very low, on the order of the experimental error.*

10. References

S1. Beley M, Collin JP, Ruppert R, Sauvage JP. *J Chem Soc Chem Comm*, 1315-1316 (1984).

S2. Neri G*, et al.* A, *Phys Chem Chem Phys* 17, 1562-1566 (2015).

S3. Anson, *et al.,* T, *J. Electroanal. Chem.*, 322, 325-345 (1992)

S4. Kelly C. A. *et al.*, *Inorg. Chem.* 38, 1579-1584 (1999)

S5. Beley M. *et al., J. Am. Chem. Soc.* 108, 7461-7467, (1986)

S6. Balazs G. B. *J. Electroanal. Chem.*, 361, 149-157 (1993)

S7. Meyer T. J. *et al.*, *Proc. Natl. Acad. Sci.*, 20918-20922 (2013)

S8. Kang P. *et al., Angew. Chem.,* 126, 1 – 6 (2014)
